# Supplementary material for: A Global Screen for Assembly State Changes of the Mitotic Proteome by SEC-SWATH-MS
Source: Cell Syst. 2020 Feb 26;10(2):133–155.e6. doi: 10.1016/j.cels.2020.01.001 (PMC7042714; doi:10.1016/j.cels.2020.01.001)

Q1KMD3 | HNRL2_HUMAN | HNRNPUL2 HNRPUL2
Monomer MW [kDa]: 85.105 Monomer expected elution fraction: 42

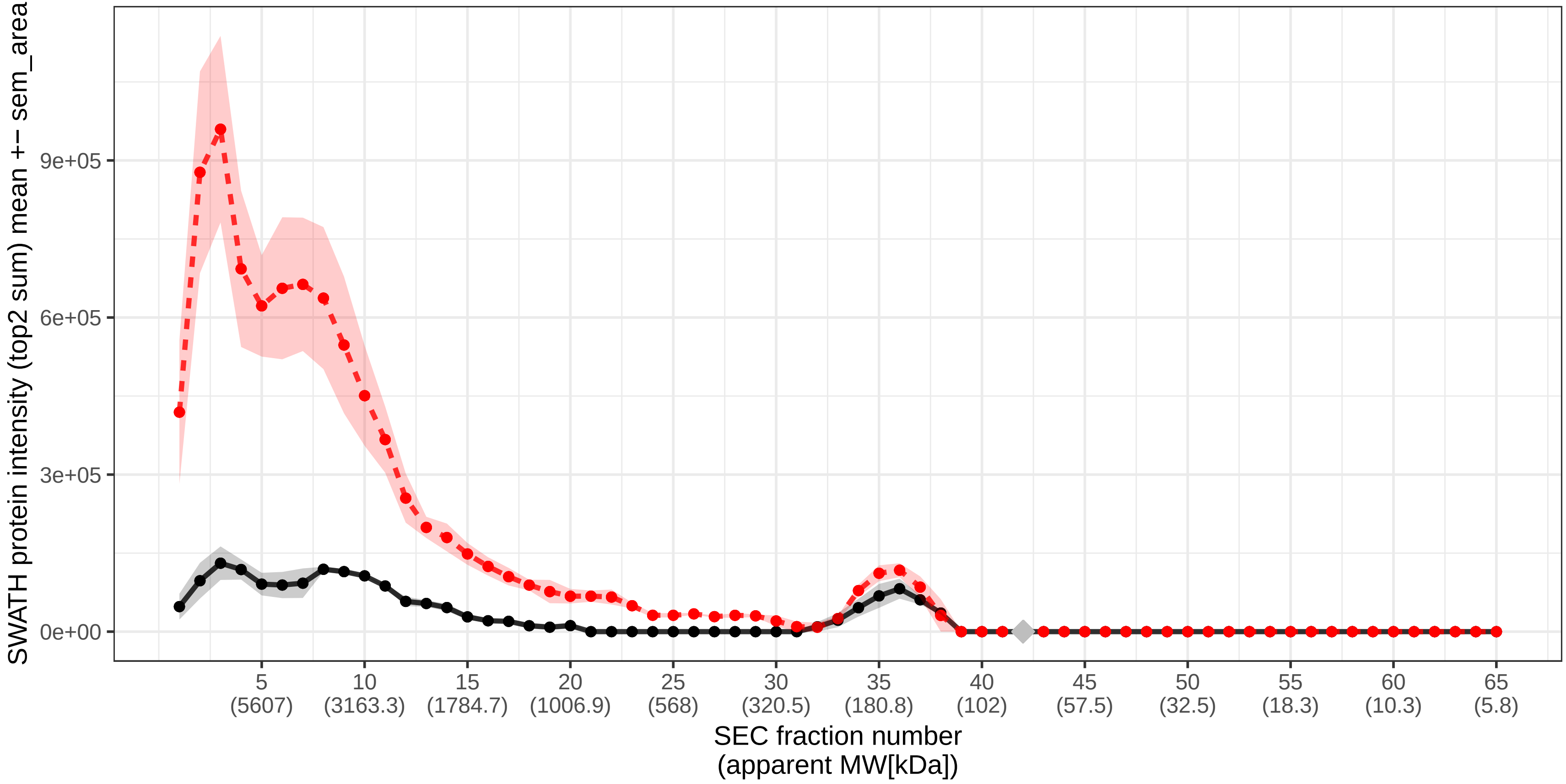

Supplement: Data S1. SEC-SWATH-MS Protein Chromatograms, Related to Figure 1 [file mmc6.zip › SECchrom_Q1KMD3_HNRL2_HUMAN_HNRNPUL2_HNRPUL2.pdf]
